# PI3K/Akt Pathway Contributes to Neurovascular Unit Protection of *Xiao-Xu-Ming* Decoction against Focal Cerebral Ischemia and Reperfusion Injury in Rats

**DOI:** 10.1155/2013/459467

**Published:** 2013-05-28

**Authors:** Rui Lan, Jun Xiang, Yong Zhang, Guo-Hua Wang, Jie Bao, Wen-Wei Li, Wen Zhang, Li-Li Xu, Ding-Fang Cai

**Affiliations:** ^1^Department of Integrative Medicine, Zhongshan Hospital, and Laboratory of Neurology, Institute of Integrative Medicine, Fudan University, Shanghai 200032, China; ^2^Longhua Hospital, Shanghai University of Traditional Chinese Medicine, Shanghai 200032, China; ^3^Guangzhou University of Traditional Chinese Medicine, Guangzhou 510006, China; ^4^Shuguang Hospital, Shanghai University of Traditional Chinese Medicine, Shanghai 200021, China

## Abstract

In the present study, we used a focal cerebral ischemia and reperfusion rat model to investigate the protective effects of *Xiao-Xu-Ming* decoction (XXMD) on neurovascular unit and to examine the role of PI3K (phosphatidylinositol 3-kinase)/Akt pathway in this protection. The cerebral ischemia was induced by 90 min of middle cerebral artery occlusion. Cerebral infarct area was measured by tetrazolium staining, and neurological function was observed at 24 h after reperfusion. DNA fragmentation assay, combined with immunofluorescence, was performed to evaluate apoptosis of neuron, astrocyte, and vascular endothelial cell which constitute neurovascular unit. The expression levels of proteins involved in PI3K/Akt pathway were detected by Western blot. The results showed that XXMD improved neurological function, decreased cerebral infarct area and neuronal damage, and attenuated cellular apoptosis in neurovascular unit, while these effects were abolished by inhibition of PI3K/Akt with LY294002. We also found that XXMD upregulated p-PDKl, p-Akt, and p-GSK3**β** expression levels, which were partly reversed by LY294002. In addition, the increases of p-PTEN and p-c-Raf expression levels on which LY294002 had no effect were also observed in response to XXMD treatment. The data indicated the protective effects of XXMD on neurovascular unit partly through the activation of PI3K/Akt pathway.

## 1. Introduction

Current biomedical research about stroke is focusing on treating neurovascular unit (NVU), and it is widely accepted that the key to effective therapy lies in restoring normal function of NVU.

NVU is a functionally and structurally interdependent multicellular complex which consists of endothelial cells, basal lamina, pericytes, astrocytes, and neurons [1], and the various components of NVU dynamically interact and act as an intricate network to maintain the homeostatic microenvironment for neuronal survival and function [2]. Amounting evidence indicates that NVU plays an important role in physiological functions and pathogenesis of many central nervous system diseases such as stroke and Alzhimer's disease [2, 3]. Despite a growing comprehension of the molecular process that causes ischemia injury to neuron, we still need to understand the whole changes of NUV after stroke such as glial cells and endothelial cells, not only neurons. 

Cerebrovasculature and parenchymal tissues are involved in the pathogenesis of stroke through the active interaction of multiple mechanisms, and ischemic penumbra as the potentially therapeutic target has become the focal point of stroke research. Apoptosis, a special form of cell death, occurs in this highly complex process, and neuronal fate after ischemia is dependent on the balance between apoptotic and survival signals. Evidence has been presented that apoptosis appears in the peripheral penumbra of ischemia [4, 5] and PI3K/Akt pathway mediates neuronal survival after cerebral ischemia and reperfusion [6–8]. Phosphorylation of Akt promotes cell survival against cerebral ischemic insult by phosphorylation and subsequent inactivation of many proapoptotic proteins, such as glycogen synthase kinse 3*β* (GSK3*β*) [7], Bad [9], and Forkhead transcription factors [10]. Although there is growing evidence that PI3K/Akt pathway and neuronal survival following cerebral ischemia are closely correlated, few studies further clarify whether PI3K/Akt pathway contributes to the protection of NVU after cerebral ischemia and reperfusion. 

To date, a large body of basic studies have indicated that numerous agents or options could reduce cerebral infarct area and improve neurological deficits with animal models of stroke [11–21] and the suggested mechanisms may be associated with suppression of apoptosis [13–15, 19], inflammation [16, 17, 19], glutamate excitotoxicity [18, 21], energy deficiency [11, 20], superoxide-mediated oxidative stress [12], and promotion of angiogenesis [19] by activation of signal pathways. However, few potential drugs or strategies could eventually be applied to benefit patients with stroke. A report summarized the problems which may cause the failures of the conversion and showed that there were many discrepancies on the outcome measures, functional assessment, and discrepancies of premorbid conditions, therapeutic windows, and drug-dosing schedules between preclinical and clinical research [22]. Facing these successes and failures, more advances in the comprehension of the pathophysiology of stroke and more exploration of novel agents are needed [22], especially when the critical role of NVU in cerebral ischemia is proposed. 

Traditional Chinese medicine (TCM) which is derived from natural plants and animals may act on multiple targets, and its flexible and comprehensive treatment strategies usually generate unexpected outcomes. These characteristics of TCM coincide with the view of NVU protection. Based on this fact, TCM has become the focus of medical research [23–27]. *Xiao-Xu-Ming *decoction (XXMD), one classic TCM formula recorded by Sun Simiao of the Chinese ancient Tang Dynasty, has been widely used in the treatment of stroke. It has aroused extensively preclinical and clinical studies for its high efficacy [28–32]. Lines of evidence have shown that active components of XXMD could reduce cerebral infarct area, improve behavioral test, decrease iNOS activity, and attenuate mitochondrial dysfunction after cerebral ischemia [29, 30]. A recent study has reported that oral administration of middle (30 g/kg/day) and high (60 g/kg/day) dosages of XXMD could significantly inhibit apoptotic neuronal death and improve the spatial cognitive performances after ischemia. Besides, the serum isolated from XXMD-treated (60 g/kg/day) rats could block neuronal death in OGD model [28]. Most importantly, it has been proved that XXMD treatment could improve neurological outcome of patients with stroke in clinical research [31, 32]. However, NVU protection of XXMD has not been explored. Thus, we determined to identify whether XXMD could exert protective effects on NUV and confirm the role of PI3K/Akt pathway in NUV protection following cerebral ischemia and reperfusion.

## 2. Materials and Methods 

### 2.1. Reagents

LY294002 (PI3K inhibitor), the primary antibodies for phospho-PDK1 (p-PDK1, Ser241), total PDK1, phospho-PTEN (p-PTEN, Ser380), total PTEN, phospho-Akt (p-Akt, Ser473), total Akt, phospho-c-Raf (p-c-Raf, Ser259), total c-Raf, phospho-GSK3*β* (p-GSK3*β*, Ser9), total GSK3*β*, GAPDH, and horseradish-peroxidase- (HRP-) linked anti-rabbit antibody purchased from Cell Signaling Technology (Beverly, MA, USA) were used for Western blot analysis. Mouse monoclonal antibodies for NeuN and GFAP from Millipore (Billerica, MA, USA), CD31 from Abcam (HK, China), and Alexa Fluor 555 anti-mouse antibody from life technologies (Carlsbad, CA, USA) were applied to immunofluorescence. 

### 2.2. Preparation of XXMD

The XXMD consists of twelve crude drugs including *Herba Ephedrae, cassia twig, Radix Paeoniae Alba, Rhizome Chuanxiong, Radix Ginseng, Radix Stephaniae Tetrandrae, Radix Scutellariae, Semen Armeniacae Amarum, Radix Aconiti Praeparata, Radix Glycyrrhizae, Radix Ledebouriellae, and fresh Rhizoma Zingiberis Recens* in a ratio of 1 : 1 : 1 : 1 : 1 : 1 : 1 : 1 : 1 : 1 : 1.5 : 5. The crude drugs were purchased from Traditional Chinese Medicine Pharmacy of Zhongshan Hospital, Fudan University. XXMD was prepared as previously described with small modifications [28]. After the first decoction, conducted for 1 h in a 1 : 10 (w/v) drugs : water ratio, the suspension was filtered. Water was added for the second decoction, duration of about 1 h, followed by the third time which lasted 1 h. The gruffs were soaked in 75% ethanol for 24 h and the liquid was preserved. The filtered and mixed suspension from three decoctions was collected and centrifuged at 2000 ×g for 20 min to obtain a suspension for the following preparation. Ethanol was added slowly with fast agitation until the concentration reached 75% ethanol (v/v). The suspension and the liquid acquired from the gruffs were merged and centrifuged at 2000 ×g for 20 min then concentrated at the final concentration of 2 g/mL (w/v). The ethanol was recovered simultaneously with a rotary evaporator. Eventually, the liquid was autoclaved and stored at −20°C before administration.

### 2.3. Animals and Drug Administration

One hundred and six male Sprague-Dawley rats weighing 250–280 g (Experimental Animal Center, Zhongshan Hospital, Fudan University, China) were housed in groups of four with free access to food and water and maintained in temperature (22 ± 2°C) and humidity-controlled (55 ± 5%) room with 12:12 h light-dark cycle. Prior to experimental manipulation, rats were handled daily for 3 days.

We usually apply the water extract of XXMD in humans and the common human daily dosage of XXMD is 165 g/75 kg in body weight. According to the formula, *d*_rat_ = *d*_human_ × 0.71/0.11 [33], the common dosage of XXMD in rat should be 14.2 g/kg/day. As the rat's drug tolerance is higher than that of human [33], we therefore selected 15, 30, and 60 g/kg/day as low, medium, and high dosages, respectively, to investigate the effects of different dosages of XXMD on focal cerebral ischemia and reperfusion injury in the preliminary experiments. Comparing all results, we finally chose 60 g/kg/day, as the optimal dose, to further investigate the underlying mechanisms, because it is better than others in drug effect which is probably due to the higher concentration of bioactivity of XXMD at a high dosage. Moreover, the dosages were referred to previous study [28]. In the study, rats were randomly divided into five groups: sham control (Sham) group, ischemia and reperfusion (I/R) group, ischemia and reperfusion plus XXMD (60 g/kg/day, XXMD60) group, ischemia and reperfusion plus XXMD (60 g/kg/day), and LY294002 (XXMD60 + LY294002) group, ischemia and reperfusion plus XXMD (60 g/kg/day), and the solution of dimethyl sulfoxide and ethanol (XXMD60 + vehicle) group. 

For drug treatment, the rats in XXMD-treated groups were orally administered with XXMD (60 g/kg/day) and others in the Sham, I/R groups were given the same volume of distilled water for 3 days before cerebral ischemic surgery. The drug administrations were performed twice a day at 8:00 and 18:00 and continued until animals were sacrificed. The above procedures were carried out by one investigator who was aware of the animal grouping but did not participate in the sequent investigations. All experimental protocols and animal handling procedures were approved by the Animal Care and Use Committee (ACUC) of Fudan University, and were consistent with the National Institutes of Health Guide for the Care and Use of laboratory Animals.

### 2.4. Focal Cerebral Ischemia and Reperfusion

Focal cerebral ischemia and reperfusion were performed as described previously [34]. Briefly, rats were anesthetized with 10% chloral hydrate (350 mg/kg, intraperitoneal, i.p.), and the left common carotid artery (CCA), the external carotid artery (ECA), and the internal carotid artery (ICA) were exposed. A length of 3.0 monofilament nylon suture (18.5–19.5 mm) with its rounded tip was advanced from the ECA into the lumen of the ICA until it blocked the origin of the middle cerebral artery (MCA). Reperfusion was initiated by withdrawal of the suture until the tip cleared the lumen of the ECA after 90 min of occlusion. In the present study, the rats in the I/R and XXMD-treated groups were subjected to cerebral ischemia followed by reperfusion. Sham-operated animals underwent the same surgical procedure, but the suture was not introduced.

It should be noted that there are several technical factors in the MCAO rat model which may affect cerebral infarct area, such as physical differences in the employed monofilament suture, the depth of the insertion, and accidental premature reperfusion [35–38]. In order to avoid the occurrence of the above technical factors, the employed monofilament sutures were unified and purchased from Beijing Sunbio Biotech Co., Ltd. (Beijing, China). One adequately trained operator who was blind to the experimental design performed the focal cerebral ischemia and reperfusion induction using the standardized surgical and technical procedures to generate reproducible ischemic lesions in animal experiments.

### 2.5. Intracerebral Ventricular Injection

**Figure 1:**
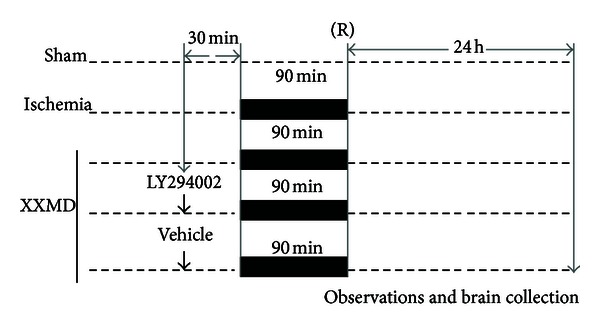
Schematic representation of the experimental protocols. Rats were randomly divided into five groups. Sham group in which the left ICA was exposed surgically but not subjected to occlusion. In the I/R and XXMD-treated groups, focal cerebral ischemia was induced in rats by MCAO for 90 min followed by reperfusion for 24 h as indicated in this figure. In the XXMD-treated group, the rats were administered with XXMD for 3 days before MCAO. In some cases, XXMD-treated rats received by intracerebroventricular injection of either the PI3K inhibitor LY294002 or vehicle 30 min prior to ischemic induction. As shown in this figure, neurological functions were observed and brains were collected at 24 h after reperfusion for TTC staining, histological analyses, TUNEL staining, immunofluorescence staining, and Western blot. Sham, sham control; ICA, internal carotid artery; I/R, ischemia and reperfusion; XXMD, *Xiao-Xu-Ming* decoction; MCAO, middle cerebral artery occlusion; R, reperfusion after cerebral ischemia.

 To investigate the function of PI3K after cerebral ischemia and reperfusion, several rats in XXMD-treated group were pretreated with LY294002, the PI3K special inhibitor, as described previously [39] with small modifications. LY294002 was dissolved in dimethyl sulfoxide (DMSO) and ethanol (ETOH) to make a final concentration of 10 mM before the operation. The preparation of LY294002 and vehicle was performed by the same investigator who was responsible for the drug treatments. Rats were securely placed into the stereotaxic device under 10% chloral hydrate (350 mg/kg, i.p.) anesthesia with bregma and lambda at a horizontal level. The skulls were exposed and determined the coordinates: anteroposterior (AP): −0.8 mm from bregma; mediolateral (ML): 1.4 mm from midline in the left side; dorsoventral (DV): −3.6 mm from skull, adapted from Paxinos and Watson [40]. A 10 *μ*L of LY294002 solution or the small amount of vehicle (DMAO + ETOH) was injected intracerebroventricularly on the ischemic side at 30 min before operation. The experimental design for the present study was shown in Figure 1.

### 2.6. Neurological Deficits Score

Examination of neurological deficits was performed at 24 h after reperfusion by one investigator who was unaware of the experiment design. The neurological deficits were scored on a four-point scale described by Hara et al. [41] with a minor modification as follows: 0 indicated no neurological deficit; 1, mild focal neurological deficit (animal showed forelimb flexion); 2, moderate focal neurological deficit (decreased resistance to lateral push and forelimb flexion without circling); 3, severe focal deficit (decreased resistance to lateral push and forelimb flexion with circling). In the present study, the rats subjected to MCAO without any detectable neurological deficits were excluded from the following investigations and analyses to exclude operative failures.

### 2.7. Cerebral Infarct Area

Cerebral infarct area was measured by 2,3,5-triphenyl tetrazolium chloride (TTC; Sigma-Aldrich, St. Louis, MO, USA) staining. Rats were killed at 24 h after reperfusion and perfused transcardially with normal saline. Brains were sectioned into six coronal slices from rostal to caudal and stained with 1% TTC staining at 37°C for 20 min away from light. Then the brain tissues were differentiated according to white-colored infarct area and red-purple noninfarct area. Cerebral infarct areas were calculated using microscope image-analysis software (Image-Proplus, USA) according to the following formula: (contralateral hemisphere area − (ipsilateral hemisphere area − infarct area)/contralateral hemisphere area) × 100%.

### 2.8. General Histology

At 24 h after reperfusion, the rats were deep anesthetized with 10% chloral hydrate (350 mg/kg, i.p.) and perfused transcardially with 200 mL normal saline followed by 4% paraformaldehyde (0.1 M phosphate buffered saline, pH 7.4). The brains were removed and postfixed in the same fixative for 24 h at 4°C. After dehydration in graded ethanol and xylene, the brains were embedded in paraffin and sectioned into slices of 5 *μ*m on a rotary microtome. To assess brain injury, Nissl staining was performed with toluidine blue. Briefly, slices were dewaxed, dehydrated, and stained with 1% toluidine blue (Sigma-Aldrich, St. Louis, MO, USA) at 50°C for 30 min. Then the slices were rinsed and cleared in graded ethanol and xylene and coverslipped under permount. The photomicrographs were captured with a light microscope. And the number of intact cells in penumbra of ischemic cortex was counted throughout five lesion regions randomly.

### 2.9. Immunofluorescence and TUNEL Staining

In order to detect the extent of DNA fragmentation of different cells in NVU, double staining of TUNEL and immunofluorescence labeling with different antibodies were performed and the positive cells were counted. In this study, we chose anti-NeuN, anti-GFAP, and anti-CD31 antibodies, the markers of neuron, astrocyte, and vascular endothelial cell, combined with TUNEL staining to evaluate apoptosis and its location, and to further clarify the roles of multiple cells in NVU after ischemia and reperfusion.

At 24 h after reperfusion, rats were deep anesthetized and the brains were rapidly removed and cut into slices of 10 *μ*m. We performed immunofluorescence labeling with anti-NeuN, anti-GFAP, and anti-CD31 antibodies and TUNEL staining with an In Situ Cell Death Detecting Kit (Roche Diagnostics GmbH, Penzberg, Germany), respectively, according to the method described by Zhou et al. [42] and the manufacturer's protocol. Briefly, the brain slices were blocked with goat serum for 1 h and incubated with primary antibody at 4°C overnight. The slices were washed with PBS for 5 min × 3 and incubated with secondary antibodies for 60 min (light shielded) at 37°C. Then the slices were stained with DAPI (Beyotime, Haimen, Jiangsu, china) for 15 min. For TUNEL staining, the slices were incubated with TUNEL reaction mixture in a dark humidified chamber at 37°C for 1 h, followed by a final wash for 10 min × 3 with PBS and then covered with glycerine. The captured images were viewed with a fluorescence microscope (Olympus/BX51, Tokyo, Japan). TUNEL positive cells and double stained cells of different groups were counted throughout five lesion regions randomly in penumbra of ischemic cortex.

### 2.10. Western Blot Analysis

 Western blots were used to evaluate expression levels of proteins involved in PI3K/Akt pathway at 24 h after reperfusion. Whole cell protein of ischemic penumbra was extracted from the fresh brain. Equal volumes and qualities of protein solutions were separated by electrophoresis of appropriate concentrations of polyacrylamide gels and transferred to the poly-vinylidnene fluoride membranes (Millipore, Bedford, MA, USA). Then the membranes were placed in 5% skim milk, prepared with Tris-buffered saline with 0.1% Tween-20 (TBST) to block nonspecific binding, and incubated with primary antibodies at 4°C overnight. In the following 2 h, the membranes were incubated with horse-radish peroxidase-conjugated secondary anti-rabbit antibody after washing with TBST for 10 min × 3. Eventually, the targeted antigens were detected by standard chemical luminescence methods (Millipore, Bedford, MA, USA) with Fluor Chem FC 2 gel imaging system (Alpha Innotech, Santa Clara, CA, USA). GAPDH protein was used as a loading control. Phosphorylation levels of the targeted proteins were analyzed by total levels of corresponding proteins. Western blots were duplicated with three independent sets. Band intensities were measured with Quantity One software (Bio-Rad Laboratories, Hercules, CA, USA).

### 2.11. Statistical Analysis

When all the data have been collected, the animal grouping was released. Data are presented as the means ± standard error of the mean (SEM). Statistical significance was determined by one-way ANOVA followed by Tukey's multiple comparison test or unpaired Student's *t*-tests using SPSS 11.5 for Windows (Chicago, IL, USA). All were considered statistically significant for *P* values < 0.05. 

## 3. Results

### 3.1. Effects of XXMD on Cerebral Infarct Area and Neurological Deficits

The occlusion for 90 min followed by reperfusion for 24 h led to an infarct area and marked neurological deficits in rats. The results indicated that XXMD treatment resulted in a significant reduction in infarct area compared to the I/R group (21.75 ± 1.93% and 41.50 ± 2.63%, resp.). In addition, the rats subjected to cerebral ischemia and reperfusion showed noticeable neurological deficits which were markedly attenuated by XXMD treatment, while rats in the sham group performed normally after operation. 

**Figure 2:**
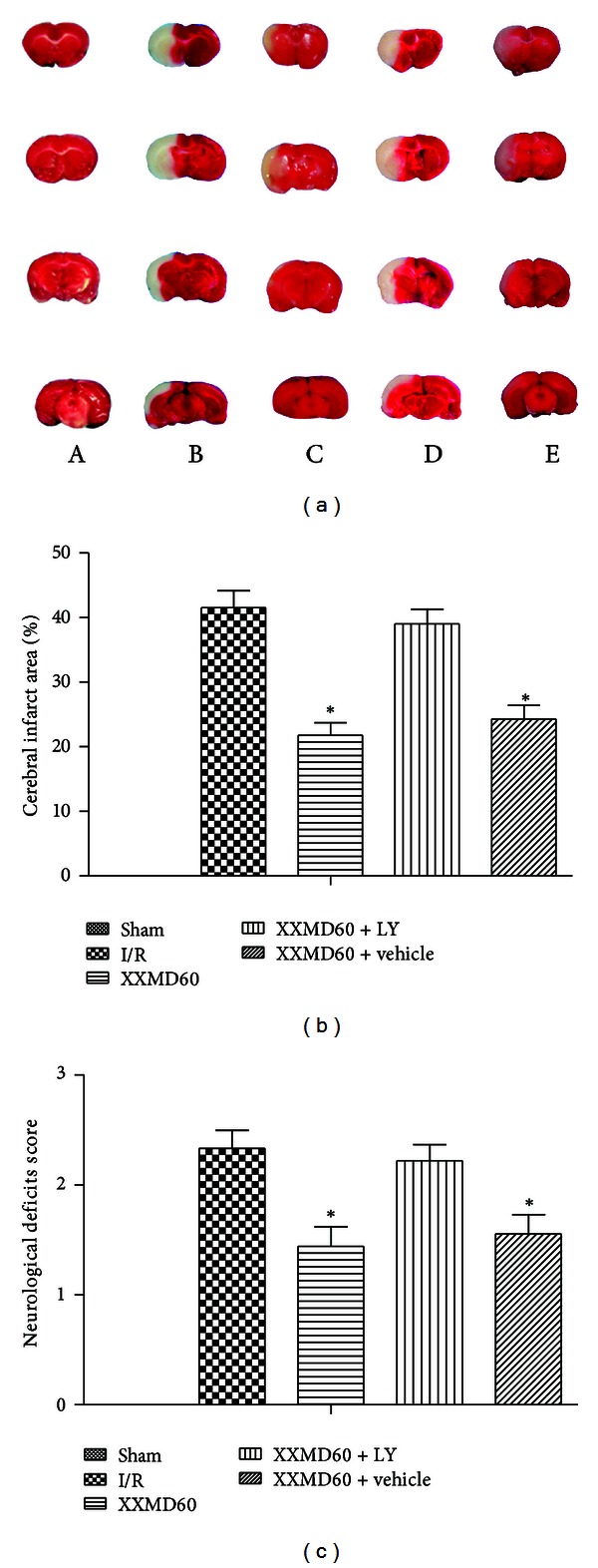
Cerebral infarct area and neurological deficits score at 24 h after reperfusion. (a) Representative images of brain slices stained with 1% TTC. The infarct area appeared white, whereas noninfarct areas were stained red purple. (b) Quantitative analysis of cerebral infarct areas. XXMD treatment reduced infarct area and LY294002 partially abolished the reduction in infarct area induced by XXMD treatment. (c) Effect of XXMD on neurological deficits score. XXMD treatment significantly improved neurological deficits score and LY294002 reversed effects of XXMD treatment. A, sham group; B, I/R group; C, XXMD60 group; D, XXMD60 + LY294002 group; E, XXMD60 + vehicle group; LY, LY294002. Data are reported as the means ± SEM. *n* = 5; *P < 0.05 versus the I/R group.

To evaluate the contribution of PI3K/Akt pathway to cerebral infarct area and neurological deficits, we investigated the effects of inhibiting PI3K with the specific inhibitor LY294002 on cerebral infarct area and neurological function at 24 h after reperfusion. Larger infarct area was observed in LY294002-treated rats (39.01 ± 2.27%) compared with the rats in the XXMD60 group. Additionally, we found a smaller infarct area in the XXMD60 + vehicle group (24.25 ± 2.23%) compared with the I/R group (Figures 2(a) and 2(b)). Likewise, LY294002 blocked the effects of XXMD60 on neurological deficits (Figure 2(c)). These findings were consistent with the hypothesis that XXMD-induced neuroprotection required the activation of PI3K/Akt pathway.

### 3.2. Effects of XXMD on Neuronal Injury

**Figure 3:**
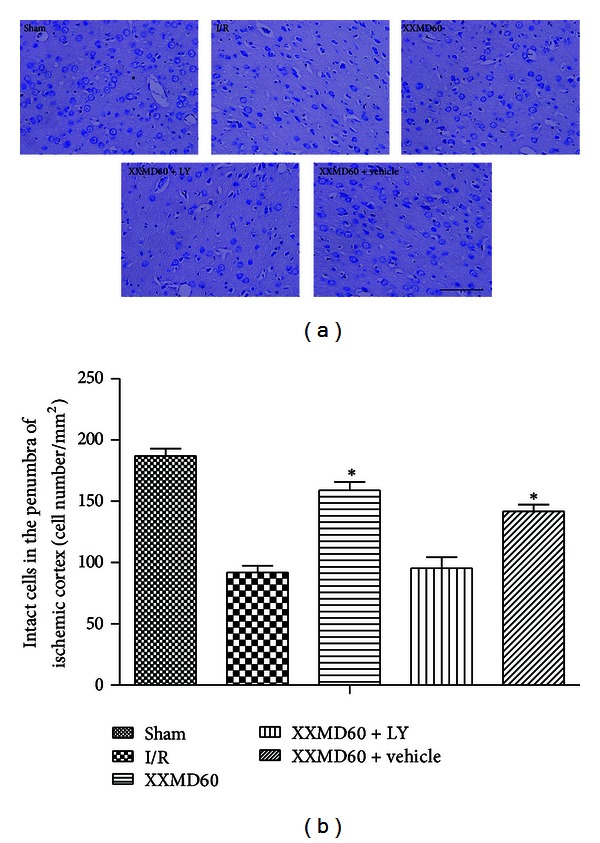
Representative images of Nissl staining and analysis of intact cells in different groups at 24 h after reperfusion. (a) The images of Nissl staining. Normal neurons were arranged orderly and had normal morphology with intact structure, abundant cytoplasm, and clear nucleolus in sham group. Most neurons in the damaged area appeared shrunken and deep stained in the I/R group. XXMD treatment improved the ischemic changes, which were blocked by LY294002. (b) Analysis of intact cells in penumbra of ischemic area. The number of intact cells in XXMD60 group was significantly higher than that in the I/R group. LY, LY294002. Scale bar = 50 *μ*m. Data are reported as the means ± SEM. *n* = 6; *P < 0.05 versus the I/R group.

Nissl staining showed neuronal injury in penumbra of ischemic cortex at 24 h after reperfusion. The images showed that most cells with intercellular space enlarged were shrunk in the ischemic cortex and had deep color staining indicative of injury in the I/R group. However, these characteristic changes were not observed in the sham group and were improved by XXMD treatment. Interestingly, LY294002 blocked the effect of XXMD on neuronal injury. Furthermore, there were more intact cells in the ischemic cortex of the XXMD-treated rats in the absence of LY294002 compared to the I/R group (Figure 3).

### 3.3. Effects of XXMD on Neuron Apoptosis

**Figure 4:**
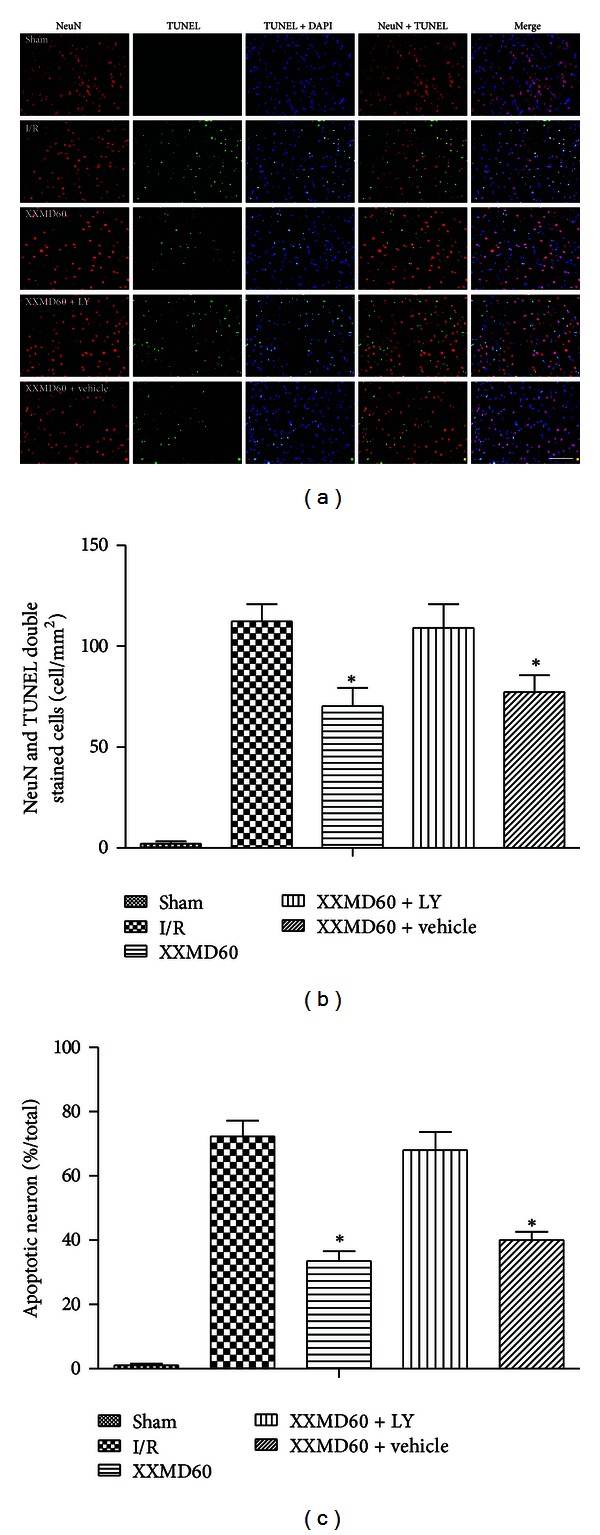
Representative images of neuron apoptosis of different groups in penumbra of ischemic cortex at 24 h after reperfusion. (a) Representative photomicrographs of immunofluorescence labeling with NeuN (red) and TUNEL (green) double staining. Nuclei were counterstained with DAPI (blue), and collocation of green and blue indicated TUNEL positive cell. At 24 h after reperfusion, a significant number of TUNEL-positive cells were observed in ischemic cortex of the I/R group and were strongly attenuated by XXMD treatment. (b) Quantification of apoptotic neurons. TUNEL and NeuN double stained cells (yellow) indicated the apoptotic neurons which were significantly induced by reperfusion and decreased by XXMD treatment. (c) Percentage of apoptotic neuron in NeuN positive cell. Percentage of apoptotic neuron was increased after stroke with reperfusion and was markedly reduced by XXMD treatment. However, neuron apoptosis was worsened by treatment with LY294002. LY, LY294002. Scale bar = 50 *μ*m. Data are reported as the means ± SEM. *n* = 6; *P < 0.05 versus the I/R group.

Double staining of TUNEL and immunofluorescence labeling with NeuN were performed to evaluate neuron apoptosis. As shown in Figure 4, XXMD strongly protected against neuron apoptosis in ischemic cortex at 24 h after reperfusion. The images of TUNEL staining revealed that 90 min of ischemia followed by reperfusion increased the number of TUNEL positive cells in ischemic cortex compared with the sham group, and XXMD markedly attenuated apoptosis, as indicated by a notable attenuation of the ischemia-induced increase in TUNEL positive cells. Additionally, the results of double staining showed that ischemia without XXMD treatment induced significant neuron apoptosis, whereas the number of TUNEL and NeuN double stained cells was markedly reduced in the XXMD60 group. However, PI3K inhibitor, LY294002, reduced neuron protection of XXMD, as indicated by a striking increase of neuron apoptosis. We further analyzed the percentage of apoptotic neurons in NeuN positive cells. The data indicated that there was a larger percentage of apoptotic cells in the I/R group. In striking contrast, we found that XXMD promoted neuronal survival, as indicated by a profound attenuation of the percentage of apoptotic neurons. However, the effects of XXMD were blocked by treatment with the PI3K inhibitor, LY294002.

### 3.4. Effects of XXMD on Astrocyte Apoptosis

**Figure 5:**
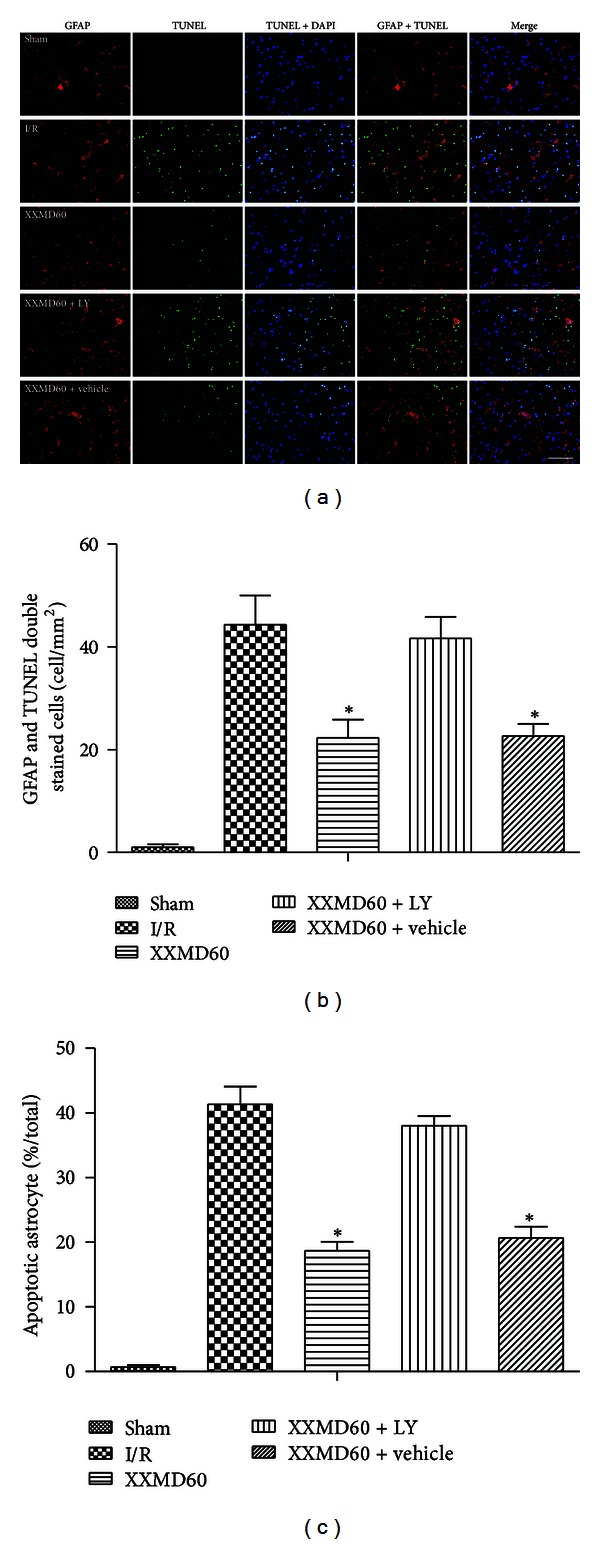
Representative images of astrocyte apoptosis in penumbra of ischemic cortex at 24 h after reperfusion. (a) Representative photomicrographs of immunofluorescence labeling with GFAP (red) and TUNEL (green) double staining. Nuclei were counterstained with DAPI (blue). And collocation of green and blue indicated TUNEL positive cell in the same view. Stroke with reperfusion notably caused TUNEL-positive cells at 24 h after reperfusion without XXMD treatment, and a decrease was found in XXMD60 group. However, LY294002 abolished the reduction. (b) Quantification of apoptotic astrocytes. TUNEL and GFAP double stained cells (yellow) indicated the apoptotic astrocytes. A marked increase in apoptotic astrocytes was found at 24 h after reperfusion and was reversed by XXMD treatment. (c) Percentage of apoptotic astrocyte in GFAP positive cell. There were more nonapoptotic astrocytes in the XXMD-treated groups in absence of LY294002 compared with the I/R group. LY, LY294002. Scale bar = 50 *μ*m. Data are reported as the means ± SEM. *n* = 6; *P < 0.05 versus the I/R group.

To comprehensively determine apoptosis of different cells in NVU, we next performed double staining for TUNEL and immunofluorescence labeling with GFAP to evaluate astrocyte apoptosis. As represented in the ischemic cortex, the number of TUNEL and GFAP double stained cells was markedly increased in penumbra of ischemic cortex at 24 h after reperfusion. These changes were largely reversed in the XXMD60 group but worsened in the presence of LY294002. In all cases, double stained cells were hardly observed in the sham group. Percentages of apoptotic astrocyte in different groups were similar with the above findings (Figure 5).

The data revealed that astrocyte, as the key component of NVU, was involved in apoptosis induced by stroke with reperfusion. However, astrocyte apoptosis was decreased by treatment with XXMD. In view of the important role of astrocyte, the results further indicated that there were more normal astrocytes which promoted neuronal survival by XXMD treatment.

### 3.5. Effects of XXMD on Vascular Endothelial Cell Apoptosis

**Figure 6:**
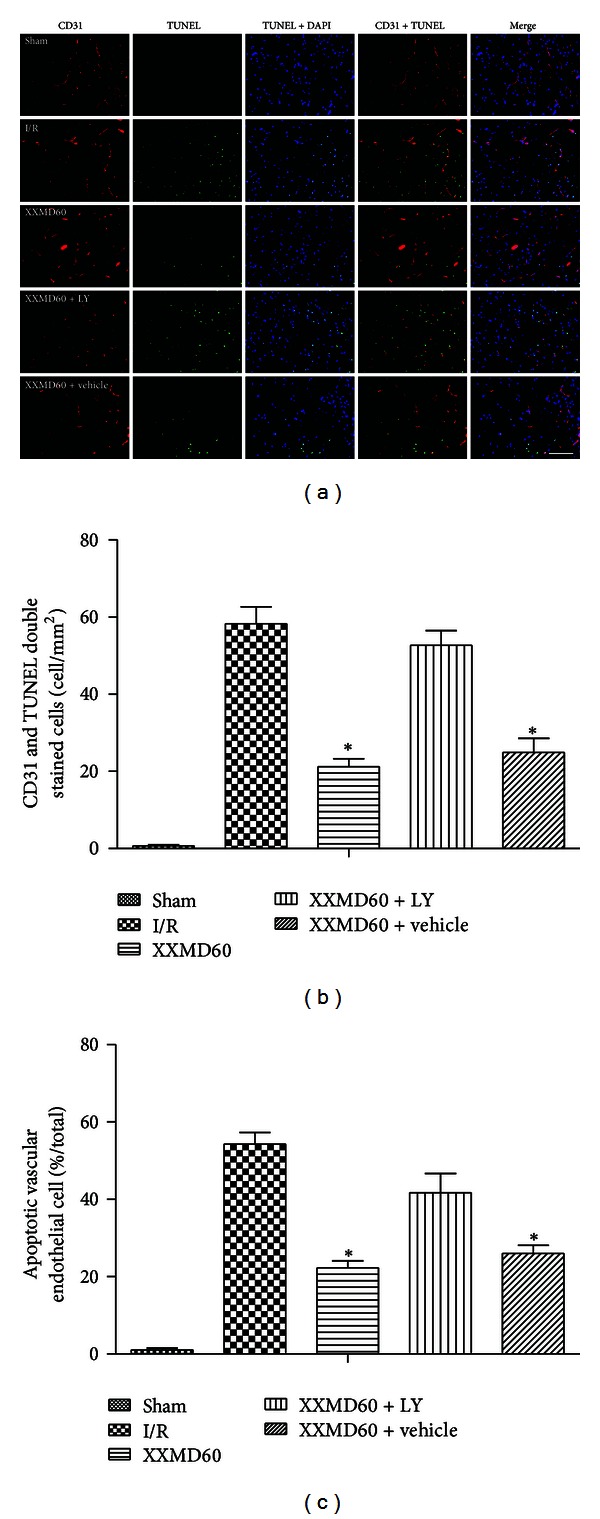
Representative images of vascular endothelial cell apoptosis in penumbra of ischemic cortex at 24 h after reperfusion. (a) Representative photomicrographs of immunofluorescence labeling with CD31 (red) and TUNEL (green) double staining. Nuclei were counterstained with DAPI (blue). And collocation of green and blue indicated TUNEL positive cell in the same view. (b) Quantification of apoptotic vascular endothelial cells. TUNEL and CD31 double stained cells (yellow) indicated the apoptotic vascular endothelial cells. Cerebral ischemia and reperfusion induced a significant increase in apoptotic vascular endothelial cell which was prevented by XXMD treatment. However, LY294002 inhibited the antiapoptotic effect of XXMD on vascular endothelial cell. (c) Percentage of apoptotic vascular endothelial cell in CD31 positive cell. There were more nonapoptotic vascular endothelial cells in the XXMD-treated groups in the absence of LY294002 compared with the I/R group. LY, LY294002. Scale bar = 50 *μ*m. Data are reported as the means ± SEM. *n* = 6; *P < 0.05 versus the I/R group.

Intriguingly, the results of CD31 and TUNEL double staining in different groups were consistent with antiapoptotic effects of XXMD on neuron and astrocyte after ischemia and reperfusion. As reperfusion occurred, vascular endothelial cell, another considerable component of NVU, was subjected to severe damage unavoidably and showed the changes of apoptosis. However, XXMD treatment significantly reduced vascular endothelial cell apoptosis which was prevented by intracerebroventricular injection of LY294002 (Figure 6).

### 3.6. Effects of XXMD on the Expression of Proteins Involved in PI3K/Akt Pathway

**Figure 7:**
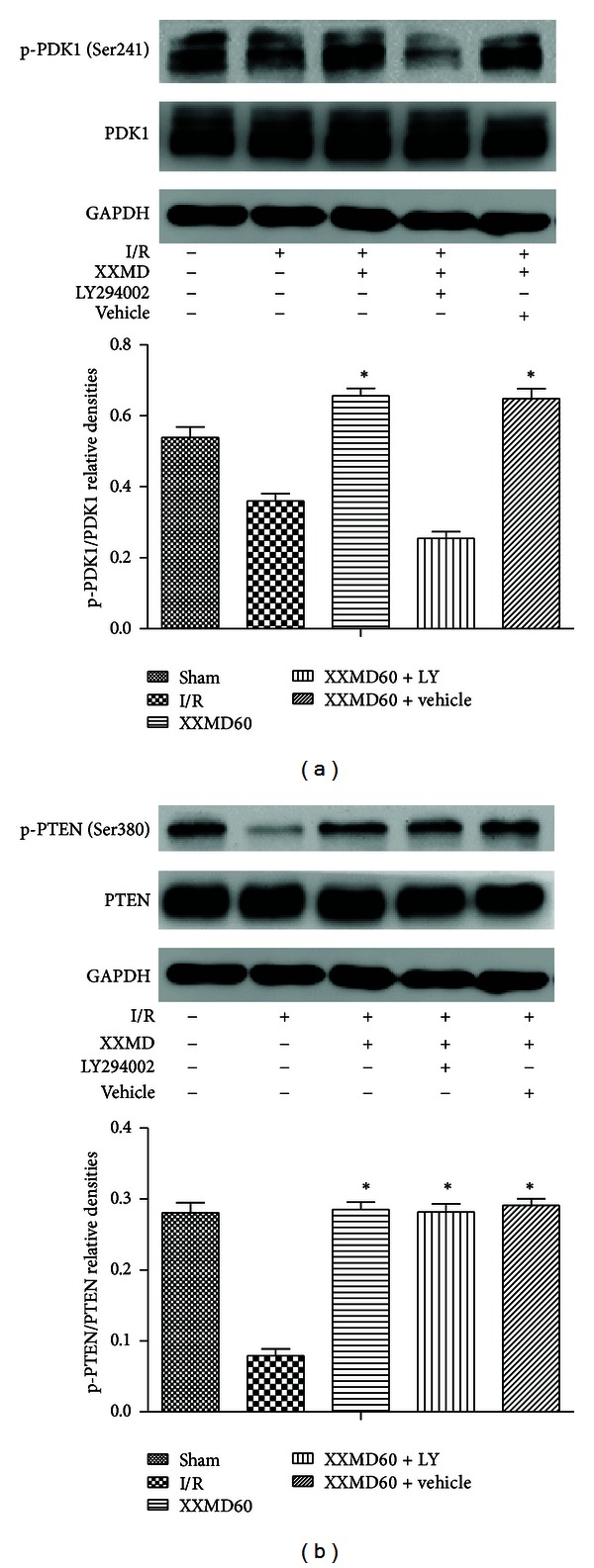
Western blot analysis of phosphorylation levels of PDK1, PTEN at 24 h after reperfusion. (a) Representative protein bands for p-PDK1 (Ser241), total PDK1, were shown along with their corresponding relatively densities. Although the levels of p-PDK1 (Ser241) were significantly decreased at 24 h after reperfusion, XXMD treatment blocked such changes. However, the effects were partly blocked by LY294002. No changes in PDK1 were detected in rats of different groups. GAPDH was used to show equal protein loading of each lane. (b) Representative protein bands for p-PTEN (Ser380), total PTEN. Relative densities demonstrated that p-PTEN (Ser380) expression levels were decreased at 24 h after ischemia. Levels of p-PTEN (Ser380) in the XXMD-treated rats were significantly higher than those in the I/R group, suggesting that XXMD treatment upregulated protein levels of p-PTEN (Ser380) after stroke with reperfusion and LY294002 had no effect on its expression. No changes in PTEN were observed in rats of different groups. Data are reported as the means ± SEM. *n* = 5; *P < 0.05 versus the I/R group.

**Figure 8:**
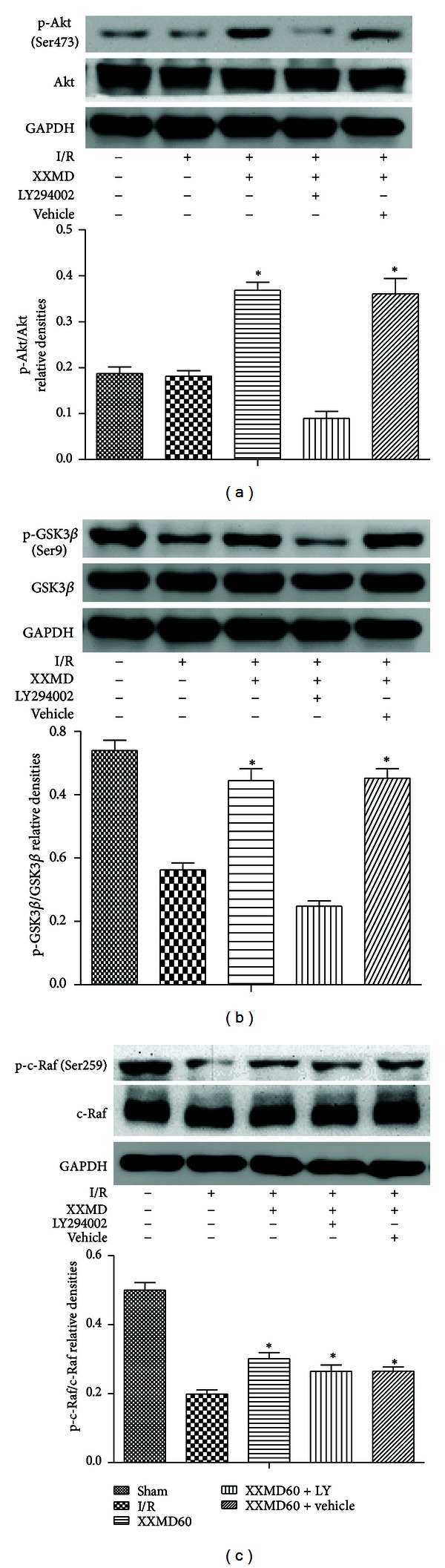
Western blot analysis of phosphorylation levels of Akt, GSK3*β*, and c-Raf at 24 h after reperfusion. (a) Representative protein bands for p-Akt (Ser473), total Akt. p-Akt (Ser473) expression levels significantly were decreased at 24 h after reperfusion, whereas XXMD treatment enhanced p-Akt (Ser473) levels and the effects could be partly reversed by PI3K inhibitor. No changes in Akt were detected in rats of different groups. GAPDH was used to show equal protein loading of each lane. (b) Representative protein bands for p-GSK3*β* (Ser9), total GSK3*β*. A decrease in p-GSK3*β* (Ser9) level was observed in the peripheral area of ischemia after reperfusion and the levels of p-GSK3*β* (Ser9) in the XXMD60 group were higher than those in the I/R group. However, inhibition of PI3K using LY294002 abolished the increase. No changes in GSK3*β* were observed in rats of different groups. (c) Representative protein bands for p-c-Raf, total c-Raf. Although p-c-Raf expression levels significantly were decreased at 24 h after reperfusion, XXMD preserved the levels of p-c-Raf. Notably, LY294002 did not significantly block the effect of XXMD on p-c-Raf. No changes in c-Raf were detected in rats of different groups. Data are reported as the means ± SEM. *n* = 5; *P < 0.05 versus the I/R group.

Since the effects of the PI3K inhibitor indicated that XXMD may exert NVU protection via PI3K/Akt signaling pathway, we further examined whether XXMD regulated PI3K/Akt pathway following focal cerebral ischemia and reperfusion. The expression levels of related proteins in the penumbra of ischemic area were detected by Western blots. As shown in Figures 7 and 8, the results revealed that ischemia and reperfusion significantly induced remarkable decreases in the expression levels of p-PDK1, p-Akt, and p-GSK3*β*, whereas XXMD treatment maintained the phosphorylation levels of these proteins, which were reversed upon pretreatment with LY294002. Changes in total levels of PDK1, Akt, and GSK3*β* were not observed in different groups at 24 h after reperfusion. Additionally, our data indicated that p-PTEN levels were decreased after ischemia and reperfusion, and XXMD treatment attenuated the declines in p-PTEN after ischemia. However, LY294002 administration had no effect on the expression of p-PTEN in the XXMD-treated rats. Another surprising finding in this study was that XXMD treatment in the absence or presence of LY294002 effectively upregulated the expression of p-c-Raf, which was decreased after cerebral ischemia and reperfusion. Moreover, the data showed that total PTEN and c-Raf levels of different groups were not significantly different. 

## 4. Discussion

Stroke, as one of the leading causes of human death and disability, has become a severe threat to human health. Although the researchers have conducted plenty of preclinical studies and obtained many striking outcomes, translating bench success to the bedside proof of efficacy and safety has been frustrating [22]. There is a pressing need to resemble the human stroke with a satisfactory and appropriate animal model and scientifically evaluate the neuroprotective strategies [22]. It is well known that middle cerebral artery occlusion (MCAO) is one main cause of human ischemic strokes [43]. MCAO model, of which the employed procedures are noninvasive and easy to perform, could mimic the pathophysiological changes in human stroke and create reproducible territory infarct of MCA. Moreover, MCAO is non-expensive and enables the investigators to monitor the physiologic parameters during the experiments and analyze the outcome measures [38]. As much, MCAO has been one of the classic methods to induce the focal cerebral ischemia and reperfusion injury and test neuroprotective agents.

In recent years, several scientists have focused on TCM, a potential and promising therapeutic approach for cerebral ischemia and investigated its efficacy and mechanism. XXMD is beneficial for the patients with stroke. A spate of studies has indicated that XXMD is prepared from twelve medical herbs containing a number of active fractions [44–46]. The effective components of XXMD such as paeoniflorin [47–49], baicalin [50–53], wogonin [54, 55], Baicalein [56–60], glycyrrhizic acid [61], and tetramethylpyrazine [62, 63] have been identified that they have antioxidant, anti-inflammatory, and antiapoptotic properties and could achieve neuroprotective effects following stroke with reperfusion. A better knowledge of the effects and underlying mechanisms of XXMD is very important to link the data from preclinical studies to clinical effects. Thus, we induced the focal cerebral ischemia and reperfusion injury with MCAO and selected the different doses of XXMD according to common human daily dose to observe the effects of XXMD on ischemic injury. Based on the preliminary results, we eventually determined the optimal dosages (60 g/kg/d) for the following exploration.

In the present study, we further investigated the NVU protective effects of XXMD after ischemia and reperfusion. The results clearly demonstrated that PI3K/Akt pathway played the crucial roles in NVU protection of XXMD against focal cerebral ischemia and reperfusion injury in rats. 

Increased attention has recently been drawn to NVU protection which has quite significant implications for cerebral ischemia and reperfusion injury. Given to its important role, we focused on evaluating apoptosis of neuron, astrocyte, and vascular endothelial cell, respectively, which were important components of NVU. The results suggested that apoptosis was a common event after ischemia and reperfusion. Although apoptosis mainly located in neurons, other components of NVU were also involved in this event, such as astrocytes and vascular endothelial cells. These results further indicated that it was necessary to pay attention to NVU protection in treatment of stroke. XXMD had multiple targets to reduce apoptosis of different cells in NVU. Moreover, obvious improvements of neurological deficits and infarct area were also observed in XXMD-treated group at 24 h after reperfusion. 

**Figure 9:**
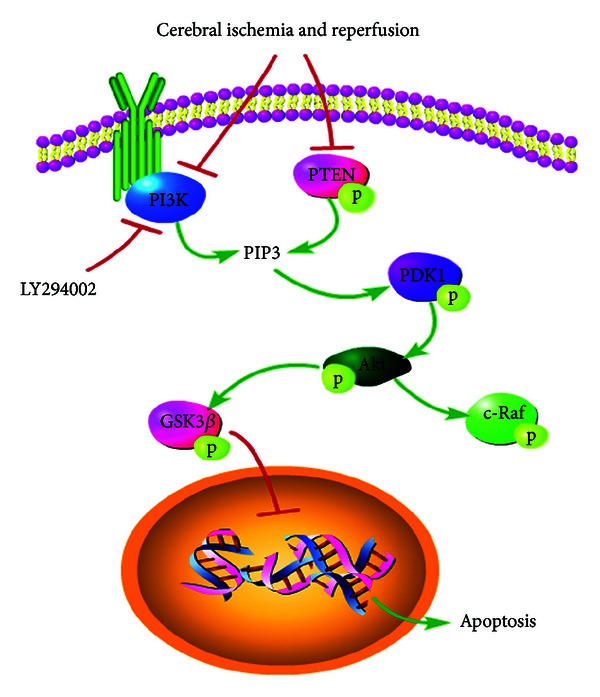
The diagram of the cell signaling pathway which was involved in NVU protection of XXMD against cerebral injury following focal cerebral ischemia and reperfusion. Reperfusion after stroke induced dysfunction of PI3K/Akt pathway and the PI3K/Akt inhibition led to dephosphorylation of PDK1, Akt, and GSK3*β* degradation, resulting in apoptosis. However, XXMD treatment inhibited apoptosis of different cells in NVU and increased expression levels of p-PTEN, p-PDK1, p-Akt, and p-GSK3*β*. Furthermore, PI3K/Akt pathway is associated with Ras/MAPK pathway. In the present study, we also found that XXMD upregulated the level of p-c-raf. Above all, all the observations in the study indicated that XXMD may exert its NVU protection partly through the activation of PI3K/Akt signaling pathway.

How did XXMD exert its NVU protection against cerebral ischemia and reperfusion injury and which signaling pathway was involved in this course? These problems need to be explored. In this study, we stressed on PI3K/Akt pathway and identified the precise and underlying mechanisms. PI3K inhibitor, LY294002, was applied to determine the role of PI3K/Akt pathway in the study, such as analyses of cerebral infarct area, neurological function, and apoptosis. All findings suggested that PI3K was involved in the effect of XXMD on NVU protection. Otherwise, the results of Western blots which were next performed showed that phosphorylation levels of PTEN, PDK1, Akt, GSK3*β*, and c-Raf were decreased in the penumbra of ischemic cortex at 24 h after reperfusion, and XXMD treatment improved phosphorylation levels of these proteins. The data also suggested that LY294002 reversed the effects of XXMD on phosphorylation levels of related proteins except for PTEN and c-Raf. These implicated that PI3K/Akt pathway contributed to neurovascular unit protection of XXMD after stroke with reperfusion (Figure 9).

Phosphoinositide dependent protein kinase-1 (PDK1) as the upstream of Akt may activate Akt by directly phosphorylating Thr308 and indirectly phosphorylating Ser473 [8, 64] and some research shows that ischemia and reperfusion induce a decrease of p-PDK1 [39]. In this study, we observed a reduction in p-PDK1 and attenuation of that effect by XXMD treatment at 24 h after reperfusion, but this effect was blocked in the presence of LY294002. Zhao et al. [39] have reported that the levels of p-Akt and p-PDK1 are discordant after reperfusion, which is consistent with our study. It may be that p-Akt is autophosphorylated or phosphorylated by other kinases [65]. However, the concrete mechanism requires additional investigation.

Plenty of reports have shown that PTEN which prevents recruitment of Akt to the membrane for phosphorylation is a negative regulator of Akt [64, 66] and downregulation of PTEN expression attenuates brain damage induced by ischemia [67–69]. However, the role of p-PTEN in cerebral ischemia remains controversial. Lee et al. [70] have suggested that a decline of p-PTEN contributes to the reduction of ischemic damage. In the study, our findings showed that p-PTEN was decreased at 24 h after reperfusion which was consistent with Zhao et al. [39] and a decline blocked by XXMD treatment. However, LY294002 did not affect the effect of XXMD on p-PTEN expression. In addition, the level of p-PTEN was coincident with p-PDK1 and p-Akt. Therefore, we proposed that increased p-PTEN expression promoted cell survival after ischemia and reperfusion. 

Akt, the serine-threonine kinase, is a key molecule in PI3K/Akt pathway and controls survival and apoptosis [71, 72] and Akt inhibits apoptosis after being phosphorylated. Accumulating evidence has suggested that the expression level of p-Akt (Ser473) could be upregulated temporarily at the onset of focal cerebral ischemia and downregulated at 24 h after reperfusion, but the expression of Akt was not significantly changed [8]. In other words, Akt plays its roles through phosphorylation rather than regulating its protein expression. In the current study, the results of Western blots showed that p-Akt was notably decreased at 24 h after reperfusion, and XXMD treatment prevented that decline but did not affect the expression of Akt. LY294002 as the special inhibitor of PI3K had striking effects on XXMD treatment that it partly blocked p-Akt expression.

Activated Akt downregulates GSK3*β* activity by phosphorylating it at Ser9 and further promotes neuronal survival [7, 73, 74]. To test downstream of Akt signaling pathway, we evaluated GSK3*β* phosphorylation of groups subjected to different administrations. The data showed a reduction in p-GSK3*β* at 24 h after reperfusion, but it was blocked by XXMD treatment. Additionally, PI3K inhibitor was involved in PI3K/Akt pathway and partly prohibited the expression of p-GSK3*β* in rats administrated with XXMD treatment. 

c-Raf, a mitogen-activated protein kinase, is a crucial target of RAS/MARK pathway. Previous studies show that Akt mediates phosphorylation of c-Raf at Ser259 and inhibits Raf kinase activity [75], thus promotes cell survival. In the current study, we also found that there was a decline of p-c-Raf expression which was reversed by XXMD treatment, but LY294002 did not change the level of p-c-Raf in XXMD-treated group. Whether RAS/MARK pathway is involved in the NUV protection of XXMD deserves further study.

Above all, we highlighted NVU protection of XXMD and elucidated the role of PI3K/Akt pathway during this course in the current study. The findings indicated that XXMD reduced cerebral infarct area and improved neurological function after ischemia and reperfusion. More importantly, we found that XXMD could protect NVU from ischemia-induced apoptosis and may exert its effects, at least in part, via the PI3K/Akt signaling pathway. Whether other pathways or mechanisms were involved in the effects of XXMD on cerebral ischemia and reperfusion injury needs our further exploration.

## 5. Conclusion

Focal cerebral ischemia and reperfusion injury were successfully induced by MCAO. Cellular apoptosis in NVU was increased in penumbra of ischemic cortex, and PI3K/Akt pathway dysfunction occurred after cerebral ischemia and reperfusion. The results indicated that treatment with XXMD reduced infarct area and cellular apoptosis and improved function outcomes after stroke. Moreover, we demonstrated that such protection may involve blunting the decreases in phosphorylation of PTEN, PDK1, Akt, GSK3*β*, and c-Raf. Taken as a whole, these effects on PI3K/Akt pathway may help explain the NVU protection of XXMD. In light of these observations, our data revealed the underlying mechanisms of XXMD treatment for stroke and provided more support for clinical applications of XXMD.
